# The Era of Climate Change Medicine—Challenges to Health Care Systems

**DOI:** 10.31486/toj.23.5033

**Published:** 2023

**Authors:** Kevin Conrad

**Affiliations:** Department of Internal Medicine, Ochsner Clinic Foundation, New Orleans, LA

*Editor's Note: With the Spring 2023 issue, we introduce a new recurring quarterly column in the Ochsner Journal. The column will focus on health care delivery and its impact on society. A particular emphasis will be placed on the role the environment, health equity, and social determinants play in health care delivery. A specific focus will be placed on the challenges of providing care to underserved populations and how health care systems are evolving to meet those needs. –R.A.*

An unprecedented joint editorial published simultaneously in 2021 in *The Lancet, New England Journal of Medicine*, and *The BMJ* stated that climate change will cause catastrophic harm to health that will be impossible to remediate unless action is taken.^1^ The editorial reported that the science is unequivocal: a 1.5 °C expected rise in temperature above the preindustrial average will cause catastrophic harm to health.^1^

A new era of climate change medicine is emerging. New diseases are being identified, existing ones are being exacerbated, and traditional health care delivery is being challenged. Climate, which has always been associated with health, is now one of the primary forces disrupting health care delivery.

Climate change is impacting health in a myriad of ways, including the health impacts of increasingly frequent extreme weather events, such as heat waves, hurricanes, and floods. These events have led to the disruption of the food supply chain, increases in zoonoses, changing patterns of vector-borne diseases, and rising mental health issues. Without intervention, the crisis threatens to undo much of the progress made in global health and poverty reduction.

Mesoamerican nephropathy, commonly called chronic kidney disease of unknown cause (CKDu), is a sentinel disease that has emerged and is directly related to exposure to increasing temperatures among field workers in Central America.^2,3^ Hot spots for CKDu are in areas most impacted by rising temperatures. In Central America, CKD—driven in part by CKDu—has now become a significant cause of hospitalization and death. CKD is now the second leading cause of death in both Nicaragua and El Salvador.^4^ The emergence of CKDu also demonstrates that marginalized populations, such as field workers, are the ones most impacted by climate change.^3,4^

Infectious diseases are also evolving as a result of climate change. The 2022 Intergovernmental Panel on Climate Change reports that the prevalence of vector-borne diseases has increased in recent decades.^5^ Warmer summers and milder temperatures have allowed pathogens to gain footholds in regions where populations have little immunity and warning systems are poorly developed.^5,6^ Rising sea temperatures also present unique problems. Harmful algal blooms, the rapid growth of algae or cyanobacteria in lakes, rivers, oceans, and bays, are increasing, potentially exposing marine life, wildlife, and humans to potent neurotoxins.^7,8^

If we are entering the era of climate change medicine, how should health care systems adapt?

First, each health care system must address its impact on climate change and its primary driver—carbon emissions. The US health care industry produces 8% of the nation's carbon emissions.^9^ A more worrisome statistic is that 10% of all smog and 9% of all particulate-related respiratory diseases can be attributed to the carbon emissions of the health care industry.^10^ One possible solution is to encourage health care systems to begin the process of becoming carbon neutral. In 2020, Kaiser Permanente became the first health care system in the United States to achieve carbon-neutral status.^11^ Gundersen Health System, a nonprofit hospital network operating in 19 counties across three Midwest states, reports that it sustainably produces more energy than it uses.^12^ Slowly, health care systems within the United States have made specific sustainability and carbon neutrality goals. Such efforts have advanced at a faster rate abroad. The United Kingdom became the first country to make a carbon neutrality pledge for its entire health care system by 2040.^13^

To assist these efforts in the United States, the Office of Climate Change and Health Equity was established in 2021 within the US Department of Health and Human Services. The aim of the office is to provide health care systems with clear metrics for assessing greenhouse gas emissions and other health care–related sustainability goals.^14^

Second, health care systems must integrate environmental information into clinical and public health practice. Robust early-warning systems that correlate climate events with disease occurrence should be developed. Examples include heat waves that may trigger heat-related nephropathy, worsening air quality that may worsen respiratory conditions, and severe weather events that cause disruption of medical services. Health systems can identify the populations most impacted by climate events and work with municipalities to reduce the impact through programs such as increased tree canopy, industrial pollution reduction, and traffic diversion. Mental first aid, a rapid post-event intervention, is also needed in response to severe weather events.

Warning systems must also monitor, in real time, the downstream effects of climate change. Such monitoring should include climate-sensitive infectious diseases such as *Vibrio vulnificus* in our waterways and vector-borne diseases such as Zika virus and dengue fever. For the first time, we are seeing climate refugees who are stressing the capacity of existing health care systems. Health care capacity should be developed to accommodate the expected increase in migration caused by land loss, crop failure, and economic pressures resulting from climate change.

Third, a health care workforce that is knowledgeable and prepared for both the physical and mental effects of extreme weather should be developed. Coronavirus disease 2019 was a global event that profoundly impacted the health care workforce.^15^ Climate change will cause similar worldwide disruptions. Health care systems are in the position to increase awareness of the impact of climate change on health within their communities, starting by educating their large workforces. Climate health literacy is an understanding of your influence on climate and climate's influence on you and society. It is essential that climate health literacy be instituted in medical schools, nursing schools, and allied health training programs. We must all speak a common language when addressing these issues. The next generation of clinicians is certainly engaged, but they need tools to handle the dynamic challenges of climate health.

Despite this multifaceted challenge, I am optimistic. Despite what may seem a futile effort, collective action is taking place.

Health care is one of the few industries that has the economic clout, the scientific basis, community engagement, and, perhaps most important, the motivation to “first do no harm” to evolve quickly. Our trusted voices are certainly needed to lead in these polarized times.

The issues of climate change are significant here in Louisiana. Since the founding of Louisiana, the climate has profoundly impacted our health. The combination of a coastal population that is susceptible to rising sea levels and extreme weather events has made Louisiana vulnerable to the effects of climate change on health and health care delivery. Our response may be seen as a laboratory and example for the rest of the country.

In Louisiana, we see the casualties of climate change firsthand and realize that time is not on our side. At a local level, coastal erosion displaces our citizens, amplifies extreme weather events, and threatens the infrastructure of our health care system. Heat waves, hurricanes, floods, and climate-driven yellow fever epidemics have defined our history. Through the years, Louisiana communities have acquired a proud resiliency, and our health care systems have always found ways to meet new challenges. Perhaps climate's impact on health is approaching a tipping point where recovery is not inevitable unless a united effort is made. Planetary health and human health have always been connected. Let's ensure our future health care systems are designed and implemented around that fact.

## References

[1] Atwoli L, Baqui AH, Benfield T, Call for emergency action to limit global temperature increases, restore biodiversity, and protect health. N Engl J Med. 2021;385(12):1134-1137. doi: 10.1056/NEJMe2113200 34491006

[2] Priyadarshani WVD, de Namor AFD, Silva SRP. Rising of a global silent killer: critical analysis of chronic kidney disease of uncertain aetiology (CKDu) worldwide and mitigation steps. Environ Geochem Health. 2022; 10.1007/s10653-022-01373-y. doi: 10.1007/s10653-022-01373-y PMC10232572 36094692

[3] De Broe ME, Vervaet BA. Is an environmental nephrotoxin the primary cause of CKDu (Mesoamerican nephropathy)? PRO. Kidney360. 2020;1(7):591-595 doi: 10.34067/KID.0003172020 35372944 PMC8815546

[4] Sorensen C, Garcia-Trabanino R. A new era of climate medicine—addressing heat-triggered renal disease. N Engl J Med. 2019;381(8):693-696. doi: 10.1056/NEJMp1907859 31433914

[5] IPCC Sixth Assessment Report. Climate change 2022: impacts, adaptations and vulnerability. Intergovernmental Panel on Climate Change. Accessed February 13, 2023. ipcc.ch/report/ar6/wg2/about/how-to-cite-this-report

[6] Caminade C, McIntyre KM, Jones AE. Impact of recent and future climate change on vector-borne diseases. Ann N Y Acad Sci. 2019;1436(1):157-173. doi: 10.1111/nyas.13950 30120891 PMC6378404

[7] Hallegraeff G, Enevoldsen H, Zingone A. Global harmful algal bloom status reporting. Harmful Algae. 2021;102:101992. doi: 10.1016/j.hal.2021.101992 33875180

[8] Patel SS, Lovko VJ, Lockey RF. Red tide: overview and clinical manifestations. J Allergy Clin Immunol Pract. 2020;8(4):1219-1223. doi: 10.1016/j.jaip.2019.10.030 31761688

[9] Richie C. Can United States healthcare become environmentally sustainable? Towards green healthcare reform. J Law Med Ethics. 2020;48(4):643-652. doi: 10.1177/1073110520979371 33404336

[10] Eckelman MJ, Sherman J. Environmental impacts of the U.S. health care system and effects on public health. PLoS One. 2016;11(6):e0157014. doi: 10.1371/journal.pone.0157014 27280706 PMC4900601

[11] Reed T. Kaiser Permanente's health system reaches carbon-neutral status. Fierce Healthcare. Published September 15, 2020. Accessed February 14, 2023. fiercehealthcare.com/hospitals/kaiser-permanente-s-heath-system-reaches-carbon-neutral-status

[12] Fabris P. Gundersen Health System says it is nation's first net-zero healthcare network. Building Design+Construction. Published January 8, 2015. Accessed February 14, 2023. bdcnetwork.com/gundersen-health-system-says-it-nations-first-net-zero-healthcare-network

[13] Jennings N, Rao M. Towards a carbon neutral NHS. BMJ. 2020;371:m3884. doi: 10.1136/bmj.m3884 33032985

[14] Balbus JM, McCannon CJ, Mataka A, Levine RL. After COP26—putting health and equity at the center of the climate movement. N Engl J Med. 2022;386(14):1295-1297. doi: 10.1056/NEJMp2118259 35363451

[15] Smallwood N, Harrex W, Rees M, Willis K, Bennett CM. COVID-19 infection and the broader impacts of the pandemic on healthcare workers. Respirology. 2022;27(6):411-426. doi: 10.1111/resp.14208 35048469

